# Endothelial Glycocalyx Disorders May Be Associated With Extended Inflammation During Endotoxemia in a Diabetic Mouse Model

**DOI:** 10.3389/fcell.2021.623582

**Published:** 2021-04-01

**Authors:** So Sampei, Hideshi Okada, Hiroyuki Tomita, Chihiro Takada, Kodai Suzuki, Takamasa Kinoshita, Ryo Kobayashi, Hirotsugu Fukuda, Yuki Kawasaki, Ayane Nishio, Hirohisa Yano, Isamu Muraki, Yohei Fukuda, Keiko Suzuki, Nagisa Miyazaki, Takatomo Watanabe, Tomoaki Doi, Takahiro Yoshida, Akio Suzuki, Shozo Yoshida, Shigeki Kushimoto, Shinji Ogura

**Affiliations:** ^1^Department of Emergency and Disaster Medicine, Gifu University Graduate School of Medicine, Gifu, Japan; ^2^Division of Emergency and Critical Care Medicine, Tohoku University Graduate School of Medicine, Sendai, Japan; ^3^Department of Tumor Pathology, Gifu University Graduate School of Medicine, Gifu, Japan; ^4^Department of Neurosurgery, Gifu University Graduate School of Medicine, Gifu, Japan; ^5^Department of Pharmacy, Gifu University Hospital, Gifu, Japan; ^6^Department of Internal Medicine, Asahi University School of Dentistry, Mizuho, Japan; ^7^Department of Clinical Laboratory, Gifu University Hospital, Gifu, Japan

**Keywords:** glycocalyx, diabetes, endothelium, inflammation, lipopolysaccharide

## Abstract

In diabetes mellitus (DM) patients, the morbidity of infectious disease is increased, and these infections can easily progress from local to systemic infection. Sepsis is a characteristic of organ failure related to microcirculation disorders resulting from endothelial cell injury, whose most frequent comorbidity in patients is DM. The aim of the present study was to evaluate the influence of infection on DM-induced microvascular damage on inflammation and pulmonary endothelial structure using an experimental endotoxemia model. Lipopolysaccharide (LPS; 15 mg/kg) was injected intraperitoneally into 10-week-old male C57BLKS/J Iar^- +^ *lepr^*db*^/lepr^*db*^* (db/db) mice and into C57BLKS/J Iar^–^m ^+^ / + *lepr*^*db*^ (db/ +) mice, which served as the littermate non-diabetic control. At 48 h after LPS administration, the survival rate of db/db mice (0%, 0/10) was markedly lower (*P* < 0.05) than that of the db/ + mice (75%, 18/24), whereas the survival rate was 100% in both groups 24 h after LPS administration. In control mice, CD11b-positive cells increased at 6 h after LPS administration; by comparison, the number of CD11b-positive cells increased gradually in db/db mice until 12 h after LPS injection. In the control group, the number of Iba-1-positive cells did not significantly increase before and at 6, 12, and 24 h after LPS injection. Conversely, Iba-1-positive cells continued to increase until 24 h after LPS administration, and this increase was significantly greater than that in the control mice. Expression of *Ext1*, *Csgalnact1*, and *Vcan* related to endothelial glycocalyx synthesis was significantly lower in db/db mice than in the control mice before LPS administration, indicating that endothelial glycocalyx synthesis is attenuated in db/db/mice. In addition, ultrastructural analysis revealed that endothelial glycocalyx was thinner in db/db mice before LPS injection. In conclusion, in db/db mice, the endothelial glycocalyx is already injured before LPS administration, and migration of inflammatory cells is both delayed and expanded. This extended inflammation may be involved in endothelial glycocalyx damage due to the attenuation of endothelial glycocalyx synthesis.

## Introduction

Endothelial disorder in patients with non-insulin-dependent diabetes mellitus (type 2 DM), which accounts for 90–95% of DM cases, is caused by chronic micro-inflammation from the early stages of diabetes (Ross, 1999). Systemic microcirculation disorder by endothelial cell injury is deeply involved in organ failure and other diseases, such as cardiovascular disease, nephropathy, retinopathy, and neuropathy (Solinas et al., 2007). In patients with type 2 DM, the morbidity of infectious disease is increased, and these infections can easily progress from local infection to systemic infection (Muller et al., 2005). Additionally, poor glycemic control in patients with type 2 DM can complicate the infection (Koh et al., 2012). Likewise, glycemic control is aggravated by infection, further increasing the severity of infection. One factor accounting for the easy contraction of infectious diseases by DM patients is endothelial disorder (Koh et al., 2012). Moreover, the most frequent comorbidity in patients with sepsis is DM (Abe et al., 2018; Kushimoto et al., 2020).

Diabetes mellitus patients account for approximately 20% of patients with sepsis (Investigators et al., 2009; Koh et al., 2012). The diagnostic criteria for sepsis include organ failure caused by several factors, including blood distribution abnormalities, heart contractility disorder, vascular hyper-permeability, endothelial injury, and decreased glomerular filtration (Singer et al., 2016). These factors are related to endothelial injury.

Vascular endothelial glycocalyx, which comprises a glycoprotein complex including syndecans, heparin sulfate, hyarulonan, and chondroitin sulfate, coats the surface of the vascular endothelium and maintains vascular homeostasis (Salmon and Satchell, 2012; Chelazzi et al., 2015). Versican, one of the core proteins of the glycocalyx, is encoded by *VCAN*. *Ext1*, *Has1*, *Has2*, and *Csgalnact1* are essential genes in the synthesis of heparan sulfate, hyaluronan, and chondroitin sulfate, respectively. Since syndecan-1 is released from the endothelium upon injury to the glycocalyx, causing its concentration in circulation to increase, syndecan-1 is useful as an endothelial glycocalyx injury marker. For instance, albumin-urea, which is a reliable marker of endothelial barrier alteration, is associated with the endothelial glycocalyx structure. Since the endothelial glycocalyx has a negative charge, the barrier of endothelial cells is destroyed and albumin flows out when the endothelial glycocalyx is injured (Adembri et al., 2011). Several previous reports have suggested associations of endothelial glycocalyx injury with severe diseases, such as acute kidney injury, chronic kidney disease, sepsis, and cardiovascular disease (Steppan et al., 2011; Padberg et al., 2014; Liborio et al., 2015; Neves et al., 2015). In addition, chronic conditions, such as diabetes (Nieuwdorp et al., 2006a, b; Broekhuizen et al., 2010), aging (Machin et al., 2018), and hypertriglyceridemia (Oda et al., 2019), injure the structure of the endothelial glycocalyx and cause degradation.

Sepsis alters neutrophil deformity in lung capillaries and subsequently causes permeability alterations and edema. In ARDS, a histologic hallmark secondary to sepsis is the recruitment of neutrophils to the lung. It has been reported that the mortality rate of granulocyte macrophage-CSFKO mice, which have only a few neutrophils and macrophages, was reduced (Spight et al., 2008). In addition, vascular endothelial glycocalyx injury was attenuated in an experimental sepsis model with a few neutrophils (Fukuta et al., 2019; Suzuki et al., 2019). In type 2 diabetes, the gene expression and phenotypic profiles of monocytes and neutrophils are altered in response to glucose (Gupta et al., 2017).

The endothelial glycocalyx regulates adhesion and migration of leukocytes, and likewise prevents the adhesion of leukocytes to endothelial cells. Therefore, it is presumed that leukocytes adhere easily to endothelial cells in injured endothelial glycocalyx in DM. However, a morphological analysis of DM-induced damage in endothelial glycocalyx has not yet been performed. In addition, it is also unknown if injured endothelial glycocalyx under DM is altered by an endotoxemic condition.

Therefore, the aim of the present study is to evaluate the influence of infection in DM-induced microvascular damage on inflammation and pulmonary endothelial structure using an experimental endotoxemia model.

## Materials and Methods

### *In vivo* Animal Studies

This study conformed to the Guide for the Care and Use of Laboratory Animals and was approved by the Institutional Animal Research Committee of Gifu University (Gifu, Japan).

C57BLKS/J Iar^–^m ^+^ / + *lepr*^*db*^ (db/ +) mice were purchased from SLC Japan Inc. (Hamamatsu, Japan). They were mated, and male C57BLKS/J Iar^−+^ *lepr^*db*^/lepr^*db*^* (db/db) mice served as a model for type 2 DM in this study. As the littermate non-diabetic control, male C57BLKS/J Iar^–^m ^+^ / + *lepr*^*db*^ (db/ +) mice were used.

After 16 h of starvation as described in previous studies (Fukuta et al., 2019; Suzuki et al., 2019), 10-week-old db/db and db/ + mice were intraperitoneally administered LPS (15 mg/kg; MilliporeSigma, Burlington, MA). Survival rates were determined at 12, 24, 36, and 48 h after LPS administration, and surviving mice were sacrificed and lung specimens collected.

### Serum Preparation and Enzyme-Linked Immunosorbent Assay

Blood samples were collected from the maxillary artery, allowed to clot at 25°C for 2 h, and centrifuged at 2,000 × *g* for 20 min at 4°C. The supernatant was collected as the serum and used for measuring IL-1β and syndecan-1 levels by enzyme-linked immunosorbent assay quantitation kits for mouse IL-1β (MLB00C; R&D Systems, Minneapolis, MN, United States) and mouse syndecan-1 (860.090.192; Diaclone, Besancon Cedex, France).

### Histopathologic Scoring

After deparaffinization, lung sections were cut (4 μm thick), counterstained with hematoxylin and eosin, and scored by a certified pathologist as follows for neutrophilic infiltration: (1) absent to rare solitary neutrophils; (2) detectable extravasated neutrophils observed as small loose cellular accumulates in one to a few airways and/or alveoli; (3) detectable extravasated neutrophils observed as loose to compact cellular accumulates in multiple to coalescing airway and/or alveoli with some effacement of lung architecture; and (4) detectable extravasated neutrophils observed as compact cellular accumulates effacing most adjacent pulmonary structures. Pulmonary edema was scored as 1, absent; 2, detectable seroproteinaceous fluid in one to a few alveoli; and 3, seroproteinaceous fluid filling alveoli in a multifocal to coalescing pattern in the lung (Suzuki et al., 2019).

### Immunohistochemistry

Lung sections were incubated with primary antibodies against the neutrophil and macrophage surface marker CD11b (ab133357; Abcam, Cambridge, United Kingdom), the vascular endothelial cell marker CD31 (DIO-310; Dianova GmbH, Hamburg, Germany), and the activated macrophage surface marker Iba-1 (019-19741; Wako Pure Chemical, Osaka, Japan). Sections were immunostained with the Vectastain Elite ABC system (Vector Laboratories, Burlingame, CA, United States) as previously described (Suzuki et al., 2019). To diminish autofluorescence, the TrueVIEW Autofluorescence Quenching Kit (Vector Laboratories, Burlingame, CA, United States) was used according to the manufacturer’s protocol. Cell counting was performed on five randomly selected high-power fields (HPF) in each section (*n* = 6).

### RNA Extraction, cDNA Synthesis, and Quantitative Real-Time PCR

RNA was extracted and purified from the lung tissues of six individual mice in each group using RNA-Bee (Tel-Test, Inc., Friendswood, TX) according to the manufacturer’s protocol. RNA concentration and integrity were assessed spectrophotometrically. RNA was reverse-transcribed using the High-Capacity cDNA Reverse Transcription Kit (Applied Biosystems, Carlsbad, CA). cDNA was a template for qRT-PCR. qRT-PCR was performed using TB Green Premix Ex Taq II (TAKARA BIO, Kusatsu, Japan) following the manufacturer’s protocol on a Thermal Cycler Dice TP 990 machine (TAKARA BIO, Kusatsu Japan). The PCR reaction conditions were 50°C for 2 min, 95°C for 10 min, and 40 cycles of 95°C for 15 s and 60°C for 1 min. The relative quantification of each transcript (*SDC1*, *Has1, Has2, Csgalnact1, Ext1*, and *VCAN*) was determined by setting the threshold cycle (Ct) for each sample to reflect the cycle number at which the fluorescence generated within the reaction crossed the threshold level chosen as a point when the amplification was in an exponential phase. *GAPDH* was the loading control. The function 2ΔCt was used to determine relative abundance differences, where ΔCt is the difference in Ct values between the compared samples. Primers used in the PCR reactions are provided in Supplementary Table 1.

### Scoring of Lectin Staining Intensity

For quantitative analysis of glycocalyx injury, scoring of *Lycopersicon esculentum* lectin (tomato lectin, B-1025-5; Vector Laboratories, Burlingame, CA, United States) staining intensity was performed using a confocal fluorescence microscope (BZ-X810, Keyence, Osaka, Japan) and ImageJ software (Olympus Corp, Tokyo, Japan). A preferred sugar of tomato lectin is N-acetylgalactosamine, which is one of the components of glycosaminoglycan, and vascular labeling by tomato lectin is a useful method to reveal vascular patterns (Robertson et al., 2015). Tomato lectin (100 μL) was injected into the jugular vein 10 min before sacrifice. The lung of each mouse was embedded in OCT compound and frozen with liquid nitrogen. The frozen blocks were stored at −80°C. Sections of frozen tissues (5–7 μm thick) were prepared with a cryostat. The intensity of tomato lectin was scored manually in 10 HPF per sample (*n* = 6 per sample) in the focal plane.

### Electron Microscopy

Electron microscopy analysis of the endothelial glycocalyx was performed as previously described (Okada et al., 2017; Ando et al., 2018; Inagawa et al., 2018). Briefly, mice were anesthetized and then perfused with a solution comprising 2% glutaraldehyde, 2% sucrose, 0.1 mol/L sodium cacodylate buffer (pH 7.3), and 2% lanthanum nitrate, at a steady flow rate of 1 ml/min, through a cannula placed in the left ventricle. After the mice were sacrificed, lung samples were fixed in a solution without glutaraldehyde and then washed in 2% alkaline (0.03 mol/L NaOH) sucrose solution. The freeze-fracture method was used to prepare samples for scanning electron microscopy (S-4800; Hitachi High-Technologies Global, Tokyo, Japan). To prepare samples for transmission electron microscopy (TEM), specimens were embedded in epoxy resin, and then ultrathin (90 nm) sections were generated, stained with uranyl acetate and lead citrate, and subjected to TEM analysis (HT-7700, Hitachi High-Technologies Global, Tokyo, Japan). To prepare samples for conventional electron microscopy, a fixative with 2.5% glutaraldehyde in 0.1 mol/L phosphate buffer (pH 7.4) was used, without lanthanum nitrate.

### Data Analysis

Data are presented as the mean ± SEM. A paired-samples *t*-test was used to compare the two groups, and survival data were analyzed using the log-rank test; *P* < 0.05 was considered significant. All calculations were performed using Prism software version 7.02 (GraphPad, La Jolla, CA).

## Results

### Profiles of Diabetic Mice

**FIGURE 1:**
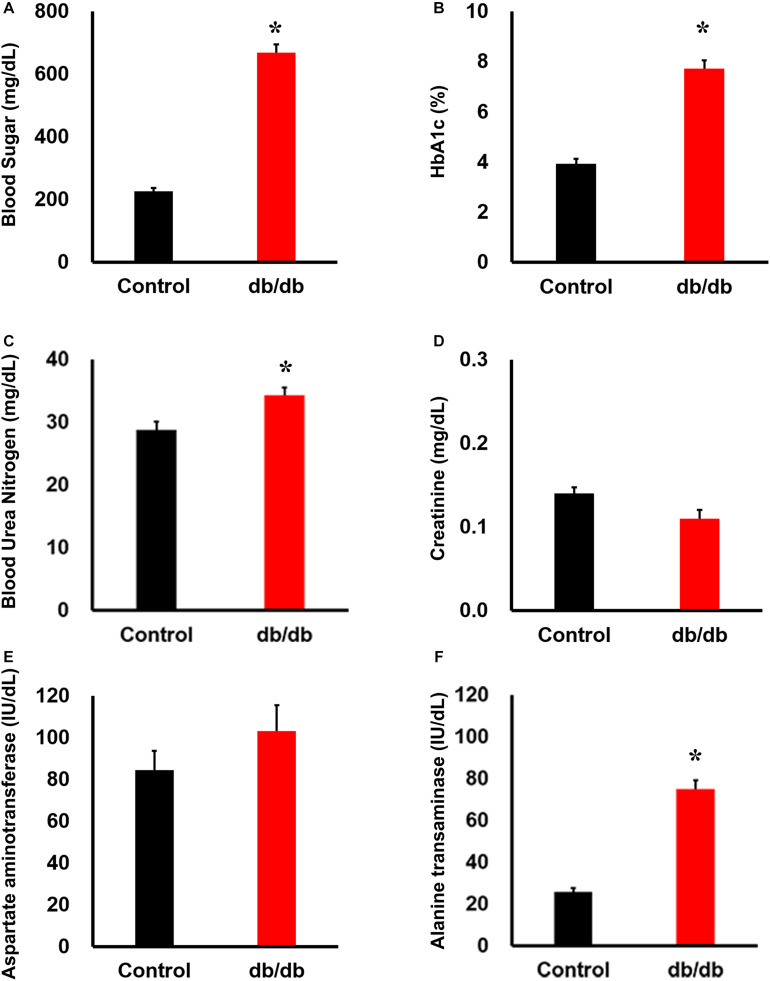
Phenotype of db/db mice under normal conditions. Serum **(A)** blood sugar, **(B)** HbA1c, **(C)** blood urea nitrogen, **(D)** creatinine, **(E)** aspartate aminotransferase, and **(F)** alanine transferase concentration: blood sugar, HbA1c, blood urea nitrogen, and alanine transferase concentrations were significantly higher in db/db mice (*n* = 6) than in the wild-type mice (*n* = 6). *, *P* < 0.05 vs. wild type.

The db/db mice had significantly greater body weight and higher plasma glucose and hemoglobin alpha1c levels than did non-diabetic db/ + mice (Figures 1A,B). Blood urea nitrogen and alanine aminotransferase levels were also higher in db/db mice than in db/ + mice, whereas creatinine and aspartate aminotransferase levels were not significantly different between the two groups (Figures 1C–F).

### Proinflammatory Cytokine Concentrations in db/db Mice Upon Lipopolysaccharide Administration

**FIGURE 2:**
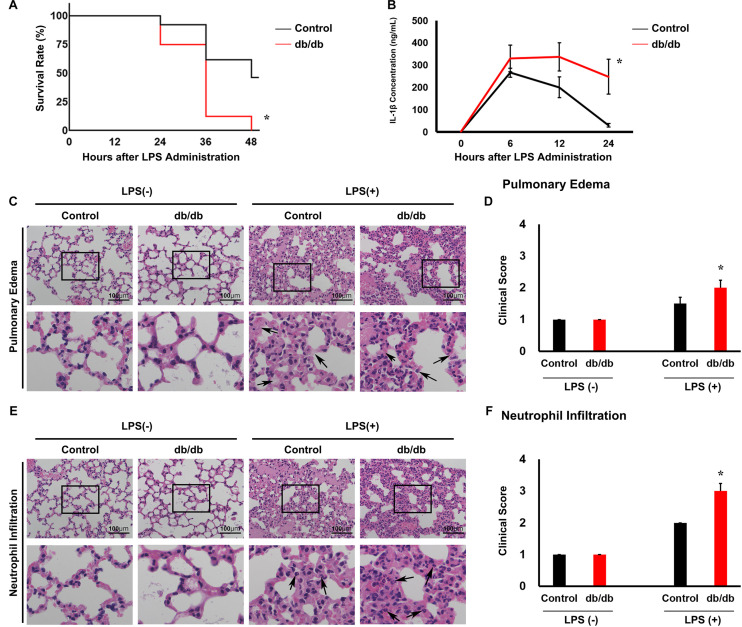
Lipopolysaccharide (LPS)-induced lung injury is accelerated in db/db mice compared with in control mice. **(A)** Kaplan–Meier survival curves for control mice (*n* = 24) and LPS-treated mice (*n* = 10). **(B)** Serum interleukin 1β (IL-1β) was measured in mice using an enzyme-linked immunosorbent assay. **(C)** Hematoxylin and eosin-stained lung tissues. Arrows indicate neutrophil infiltration. **(D)** Graphs of the histologic scoring of lung injury around the pulmonary edema. **(E)** Hematoxylin and eosin-stained lung tissues. **(F)** Arrows indicate edema. Graphs of the histologic scoring of lung injury around neutrophil filtration. *n* = 6 mice per group. *, *P* < 0.05.

To produce LPS-induced experimental endotoxemia model mice, we intraperitoneally injected 15 mg/kg LPS into 10-week-old db/db mice and littermate db/ + male mice. At 48 h after LPS administration, the survival rate of db/db mice (0%, 0/10) was markedly lower (*P* < 0.05) than the db/ + mice (75%, 18/24) (Figure 2A).

Before LPS injection, the body weight was 40.5 ± 2.4 g in db/db mice whereas it was 22.4 ± 0.6 g in the control mice. Twenty-four hours after LPS administration, the body weight was 39.2 ± 2.4 g in db/db mice whereas it was 20.5 ± 0.6 g in the control mice.

In the control mice, the serum proinflammatory cytokine IL-1β reached 266.5 ± 18.9 ng/ml at 6 h after LPS injection and 200.7 ± 42.8 ng/ml at 12 h after injection. Thereafter, the serum IL-1β levels decreased to 30.3 ± 6.7 ng/ml within 24 h after LPS injection (Figure 2B). In db/db mice, IL-1β concentration was not significantly different at 6 and 12 h after LPS administration compared with the control mice (357.0 ± 49.8 ng/ml and 337.7 ± 63.7 ng/ml, respectively). However, 24 h after LPS injection, it was 248.7 ± 85.7 ng/ml, which was significantly higher than that in control mice. However, before LPS injection, there was no significant difference between the db/db mice and control mice. This result indicated that inflammation was prolonged in db/db mice compared with the control mice.

### Lung Injury in db/db Mice Under Lipopolysaccharide Administration

To determine pulmonary injury 24 h after LPS injection, we used a scoring system (Figures 2C–G). After LPS administration, the levels of neutrophil infiltration and pulmonary edema increased compared with pre-LPS injection levels. db/db mice showed a significant increase in neutrophil infiltration and pulmonary edema compared with the control mice. These results suggest that inflammation was aggravated in db/db mice compared with the control mice.

### Inflammation Duration in db/db Mice

**FIGURE 3:**
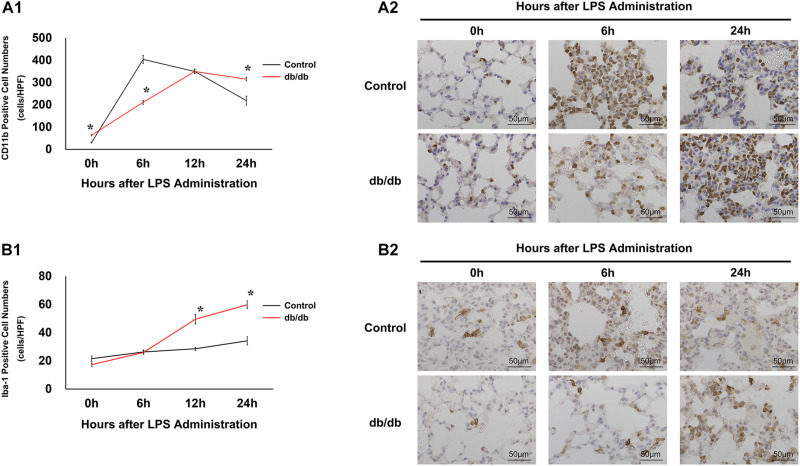
Modulation of inflammatory cells under endotoxemic conditions. **(A1)** CD11b-positive cells were quantified as numbers of cells per high-power field. **(A2)** Representative images of CD11b cells in immunostained lung specimens. **(B1)** Iba-1 positive cells were quantified as the number of cells per high-power field. **(B2)** Representative images of Iba-1 in immunostained lung samples. *, *P* < 0.05.

To examine the infiltration of inflammatory cells after LPS administration, immunohistochemistry analysis of CD11b was performed. Before LPS administration, the number of CD11b-positive cells in db/db mice is larger than in control mice. In control mice, the proportion of CD11b-positive cells increased at 6 h after LPS administration (404 ± 18 cells/HPF), and then decreased gradually. By comparison, in db/db mice, the number of CD11b-positive cells increased gradually until 12 h after LPS injection (211 ± 9 cells/HPF at 6 h, 350 ± 9 cells/HPF at 12 h after LPS administration) and gradually decreased at 24 h after LPS administration (316 ± 8 cells/HPF) (Figures 3A,B).

Furthermore, to assess activated macrophages, immunohistochemical analysis of Iba-1 was performed (Figures 3C,D). Before LPS injection, there were fewer Iba-1-positive cells in db/db mice than in control mice. In the control group, the number of Iba-1 positive cells did not significantly increase before or at 6, 12, and 24 h after LPS injection (22 ± 2, 26 ± 1, 29 ± 1, and 34 ± 3 cells/HPF, respectively). However, Iba-1-positive cells continued to increase until 24 h after LPS administration, increasing to a significantly greater extent than that in the control mice.

### Pulmonary Endothelial Glycocalyx Injury in db/db Mice

To investigate pulmonary endothelial glycocalyx injury, serum syndecan-1 concentration was measured.

**FIGURE 4:**
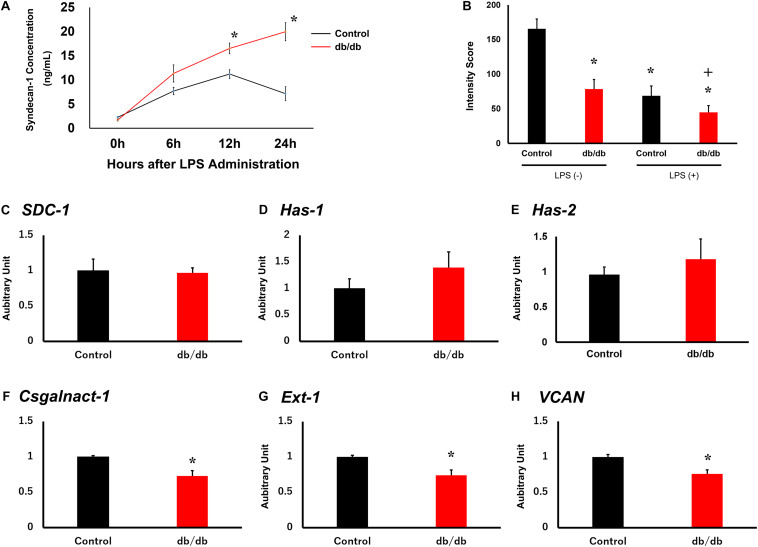
Pulmonary endothelial glycocalyx is injured in db/db mice. **(A)** Serum syndecan-1 was measured in mice using an enzyme-linked immunosorbent assay. **(B)** Tomato lectin intensity of control and db/db mouse lungs with and without LPS administration. *, *P* < 0.05 vs. non-LPS-injected control mice; +, *P* < 0.05 vs. LPS-injected control mice. **(C–H)** qRT-PCR for **(C)** *SDC1*, **(D)** *Has1*, **(E)** *Has2*, **(F)** *Csgalnact1*, **(G)** *Ext1*, and **(H)** *Vcan* in control and db/db mice under normal conditions. *, *P* < 0.05.

Serum syndecan-1 concentration in db/db mice was not significantly different from that in control mice before LPS administration (Figure 4A). In the control group, serum syndecan-1 levels reached 7.7 ± 0.8 ng/ml at 6 h after LPS injection and 11.2 ± 0.9 ng/ml at 12 h after injection. At later time points, serum syndecan-1 levels gradually decreased to 7.2 ± 1.4 ng/ml at 24 h after LPS administration. Conversely, serum syndecan-1 concentration in db/db mice continued to increase up to 24 h after LPS injection and was significantly higher than that in control mice at 12 and 24 h after LPS administration (16.5 ± 1.1 and 20.0 ± 1.9 ng/ml, respectively, vs. control mice, *P* < 0.01) (Figure 4A).

To quantitatively assess endothelial glycocalyx injury, we measured the intensity of tomato lectin staining because lectin binds to glycoproteins within the endothelial glycocalyx. Injected tomato lectin co-localize on pulmonary capillary endothelial cells (Supplementary Figure 1). In db/db mice, tomato lectin intensity was lower than in the control mice before LPS administration (Figure 4B and Supplementary Figure 2). After LPS injection, intensity score is lower in both the control and db/db mice compared with before LPS injection, and especially, it was also lower in db/db mice than in the control mice. These results suggest that injury to endothelial glycocalyx in pulmonary capillaries was aggravated in db/db mice.

### Pulmonary Endothelial Glycocalyx Synthesis in db/db Mice

To confirm endothelial glycocalyx synthesis before LPS administration, qRT-PCR was performed for syndecan-1 (*SDC1*, Figure 4C), hyaluronan synthase 1 (*HAS1*, Figure 4D), hyaluronan synthase 2 (*HAS2*, Figure 4E), chondroitin sulfate N-acetylgalactosaminyltransferase 1 (*Csgalnact1*, Figure 4F), exostosin-1 (*EXT1*, Figure 4G), and versican *(Vcan*, Figure 4H) in the control and db/db mouse groups. Results showed that the expression of *EXT1*, *Csgalnact1*, and *Vcan* in db/db mice was significantly decreased compared with that in the control mice before LPS administration.

**FIGURE 5:**
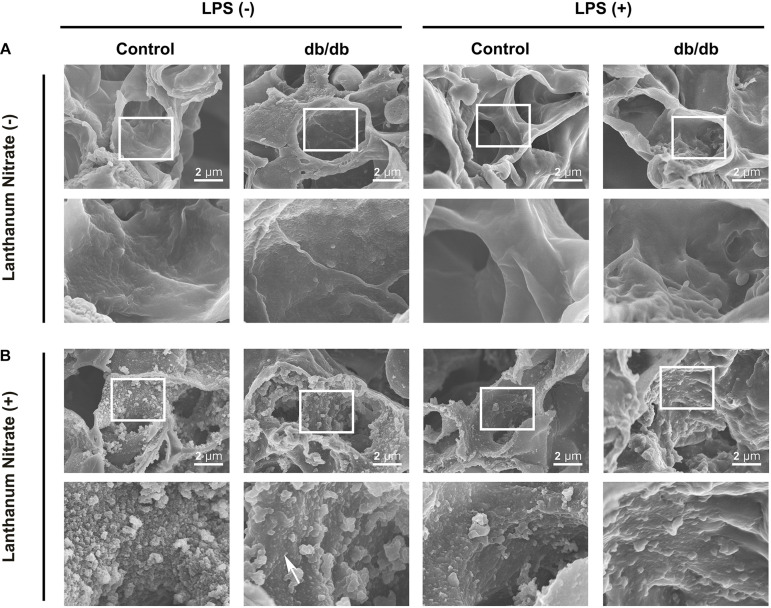
Ultrastructural imaging of pulmonary endothelial injury by SEM. **(A)** Images for samples not stained with lanthanum nitrate; the endothelial glycocalyx was not detected**. (B)** The endothelial glycocalyx was detected using lanthanum nitrate staining. Although the moss-like structure of the endothelial glycocalyx covered the surface of the vascular endothelium in the control group mice under normal conditions, the endothelial glycocalyx structure was already degraded and dispersed (white arrow) in db/db mice. After LPS injection, the endothelial glycocalyx was degraded completely in db/db mice, whereas its injury was attenuated in control mice.

The ultrastructure of the endothelium and endothelial glycocalyx was analyzed using electron microscopy. Conventional SEM results showed that pulmonary capillaries were of the continuous type, characterized by an uninterrupted endothelium and a continuous basal lamina, in control group mice before LPS administration (Figure 5A). To determine the endothelial glycocalyx structure, SEM with lanthanum staining was performed (Figure 5B). The endothelium-like structure of endothelial glycocalyx covered the surface of the vascular endothelium in the control group, under normal conditions, whereas the endothelial glycocalyx structure was thinner in db/db mice. After LPS injection, the endothelial glycocalyx was degraded completely in db/db mice, whereas its injury was attenuated in the control mice (Figure 5B).

**FIGURE 6:**
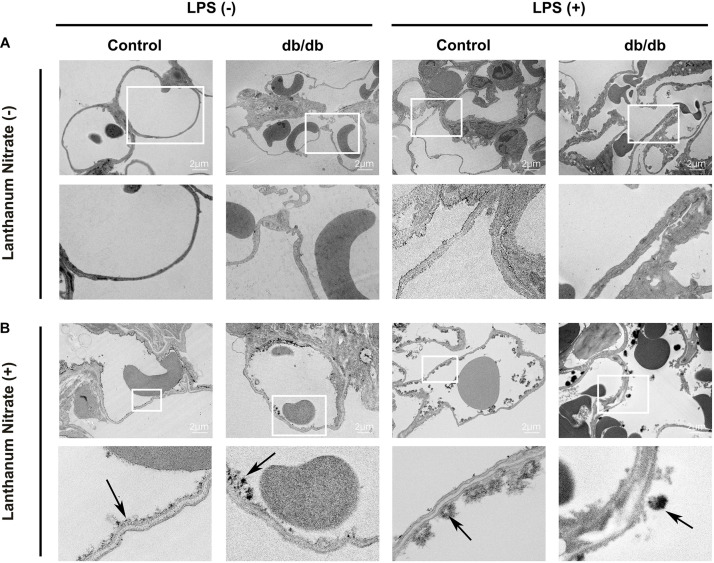
Ultrastructural imaging of pulmonary endothelial injury by transmission electron microscopy (TEM). **(A)** Images taken from samples not stained with lanthanum nitrate; the endothelial glycocalyx was not detected. The pulmonary endothelium was thin and smooth in control mice before LPS injection. In control mice after LPS injection, the endothelial wall became edematous, but the extent of this was greater in db/db mice than in control mice treated with or without LPS. **(B)** The endothelial glycocalyx was detected using lanthanum nitrate staining. The endothelial glycocalyx structure showed greater degradation in db/db mice than in control mice under normal conditions. After LPS injection, the endothelial glycocalyx formed a skip lesion in control mice, whereas the endothelial glycocalyx was more degraded in db/db mice. Black arrows indicate the endothelial glycocalyx.

Conventional TEM also revealed that the endothelium was thin and smooth in control mice before LPS injection. However, in the control mice after LPS injection, the endothelial wall became edematous, and the extent of this was greater in db/db mice than in the control mice with or without LPS (Figure 6A).

The endothelial glycocalyx structure showed greater degradation in db/db mice than in control mice, even in the absence of LPS (Figure 6B). After LPS injection, the endothelial glycocalyx caused a skip lesion in control mice, and a part of the endothelium was exposed to the vascular lumen. In db/db mice, the endothelial glycocalyx was significantly degraded than that in the control group (Figure 6B).

## Discussion

The current study showed that extended inflammation occurs in db/db mice after LPS administration. Specifically, we showed that (a) the endothelial glycocalyx layer in db/db mice was thinner than in the control mice before LPS injection, (b) the survival rate was significantly decreased in db/db mice compared with in the control group, and (c) the migration of inflammatory cells was delayed and extended in db/db mice compared with in control mice.

### Endothelial Glycocalyx Layer in db/db Mice

The endothelial glycocalyx is injured under hyperglycemic conditions (Nieuwdorp et al., 2006a, b; Hirota et al., 2020). One mechanism responsible for this may involve attenuation of glycocalyx synthesis. We found that *Ext1*, *Csgalnact1*, and *Vcan* gene expression decreased in db/db mice before LPS injection. These results suggested the possibility that endothelial glycocalyx synthesis decreased in db/db mice, although the synthesis of glycocalyx is complicated by multiple enzymatic pathways, and factors regulating its shedding include local pH and mechanical stimuli (Reitsma et al., 2007).

Previous reports have shown that endothelial glycocalyx is synthesized on endothelial cells (Mensah et al., 2017), and it was also found that endothelial cell dysregulation is involved in DM (McVeigh et al., 1992; De Vriese et al., 2000). Under hyperglycemia, glucose intake into endothelial cells is promoted via glucose transporter 1 (Mann et al., 2003), and endothelial disorder results from intracellular metabolic disorders (Wautier et al., 2001; Quagliaro et al., 2003). Likewise, insulin resistance and inflammatory cytokines can also impair the function of endothelial cells (Groop et al., 2005; Razavi Nematollahi et al., 2009; Taegtmeyer et al., 2015).

The endothelial glycocalyx is injured directly by hyperglycemia; and its synthesis may be damaged because endothelial cells under DM are exposed to hyperglycemic conditions for an extended time.

### Migration of Inflammatory Cells Is Delayed and Expanded in db/db Mice

In db/db mice, the number of CD11b-positive cells, including neutrophils and macrophages, delayed in reaching the peak compared with that in the control mouse group. After reaching the peak, the number of cells did not decrease in db/db mice, whereas cell number was significantly decreased in the lungs of control mice. This result is also supported by the result of analysis of serum proinflammatory cytokine IL-1β, which did not decrease in db/db mice compared to the control group. Meanwhile, tissue-resident macrophages, as indicated by Iba-1-positive migration to lung tissue, continued to increase in db/db mice after LPS injection, whereas there was no significant change in the control mice.

Consistent with our findings showing a delayed peak in neutrophil numbers, it was reported that neutrophil migration capability is attenuated in DM (Delamaire et al., 1997; Koh et al., 2012). However, tissue-resident macrophages may increase compensatory mechanisms, instead of neutrophils with low migration capability. Consequently, in db/db mice, extended inflammation may occur. In addition, the alteration of serum syndecan-1 concentration, serving as a marker of endothelial glycocalyx injury, was also similar to the modulation of inflammatory cells after LPS injection.

### Endothelial Glycocalyx Injury Influences Extended Inflammation

The intact glycocalyx prevents the inadvertent adhesion of platelets and leukocytes to the vascular wall (Mulivor and Lipowsky, 2002; Reitsma et al., 2007; Becker et al., 2010a, b). Specifically, the glycocalyx thickness (approximately 0.5 μm) exceeds the dimension of cellular adhesion molecules expressed on endothelial cells, such as integrins, selectins, and ICAMs, thus attenuating the interactions of these molecules with circulating blood cells (Becker et al., 2010a, b). Injury of the endothelial glycocalyx leaves the endothelial cells vulnerable to injury, and it is easy to expose the cell surface receptors to the vascular lumen, and granulocytes and platelets enable them to adhere to endothelial cells.

In db/db mice, the endothelial glycocalyx was thinner than that in the control mice before LPS administration. In addition, hyperglycemia itself stimulates ICAM expression on endothelial cells (Jafar et al., 2016). Therefore, it is thought that endothelial cells are easily injured. This phenomenon may have led to extended inflammation in the lungs of db/db mice.

Lipopolysaccharide administration causes endothelial glycocalyx injury; subsequently, the endothelial cells are damaged by pathogens and damage associated molecular patterns. It was recently reported that endothelial cells cause pyroptosis under stress (Cheng et al., 2017; Jia et al., 2019) and secrete proinflammatory cytokines, such as IL-1β. Pyroptosis is a highly inflammatory form of programed cell death that occurs most frequently upon infection with intracellular pathogens and is likely to form part of the antimicrobial response (Jorgensen and Miao, 2015). This process promotes the rapid clearance of various bacterial, viral, fungal, and protozoan infections by removing intracellular replication niches and enhancing the host’s defensive responses (Jorgensen and Miao, 2015). Although this reaction promotes inflammatory responses for host defense, excessive promotion of the inflammatory response leads to injury in the host itself. Since it is possible that pyroptosis contributes to extended inflammation in db/db mice, further study is required.

Recently, the novel coronavirus disease 2019 (COVID-19) caused by SARS-CoV-2 has become a serious concern. It is suggested that DM plays a pivotal role in COVID-19 progression. In particular, endothelial disorders, including loss of endothelial glycocalyx, may be involved in increased morbidity and mortality in diabetic patients with COVID-19 (Hayden, 2020). This phenomenon may also be explained by the extended inflammation that occurs in diabetic patients.

Notably, a limitation of this study is that it is descriptive, and further research is required to clarify the implicated mechanisms. In addition, sepsis is an exceedingly complicated disease and analysis with a simple experimental endotoxemia model may not suffice. Therefore, additional studies using a bacteremia model are required. As lanthanum binds to not only endothelial glycocalyx but also calcium-binding sites, it has been used as a calcium probe in several organs (Shaklai and Tavassoli, 1982). Therefore, lanthanum staining is not specific to glycocalyx.

## Conclusion

In conclusion, this study provided the ultrastructure of endothelial glycocalyx injury under diabetic conditions. In db/db mice, endothelial glycocalyx is already injured before LPS administration, and the migration of inflammatory cells is both delayed and expanded. This extended inflammation may be involved in endothelial glycocalyx damage due to the attenuation of endothelial glycocalyx synthesis.

## Data Availability Statement

The original contributions presented in the study are included in the article/Supplementary Material, further inquiries can be directed to the corresponding author/s.

## Ethics Statement

The animal study was reviewed and approved by the Institutional Animal Research Committee of Gifu University (Gifu, Japan). Written informed consent was obtained from the owners for the participation of their animals in this study.

## Author Contributions

SS and HO wrote the manuscript. CT, and NM performed the TEM imaging. SS, HO, KoS, HF, and YK performed the SEM imaging. CT prepared the samples for TEM imaging. SS, HO, HY, and IM prepared the samples for SEM imaging. SS, TK, and HT performed the histological assessment. SS, YF, TK, and HT performed the immunohistochemical analysis. SS, KeS, and TW performed the ELISA. SS, HY, IM, TD, TY, and SY conducted the animal studies. RK and AS performed the FACS analysis. SK and SO supervised the animal studies. HO and HT revised and edited the manuscript. All authors have read and approved the final manuscript.

## Conflict of Interest

The authors declare that the research was conducted in the absence of any commercial or financial relationships that could be construed as a potential conflict of interest.
