# Supplementary figures and images for: Endothelial Glycocalyx Disorders May Be Associated With Extended Inflammation During Endotoxemia in a Diabetic Mouse Model
Source: Front Cell Dev Biol. 2021 Apr 1;9:623582. doi: 10.3389/fcell.2021.623582 (PMC8047120; doi:10.3389/fcell.2021.623582)

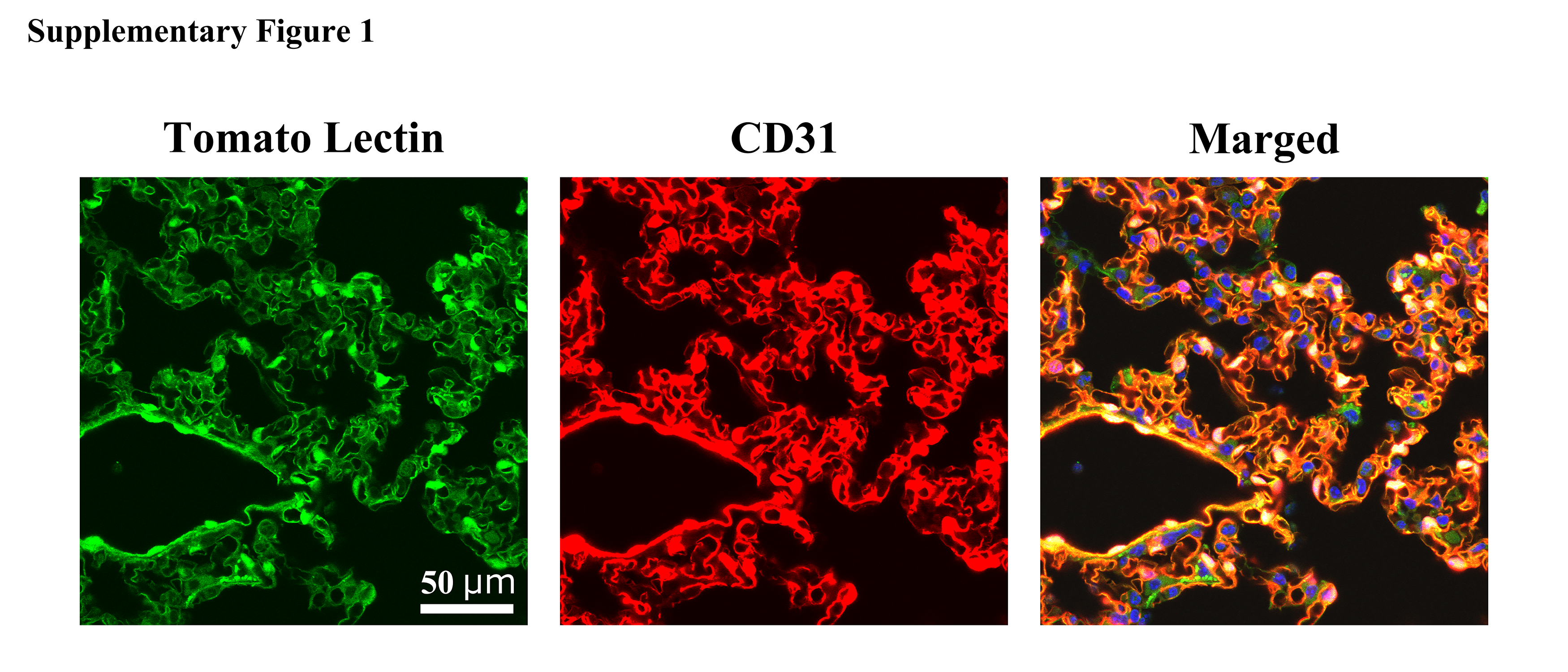

Supplement: Supplementary file 1 [file Image_1.TIF]

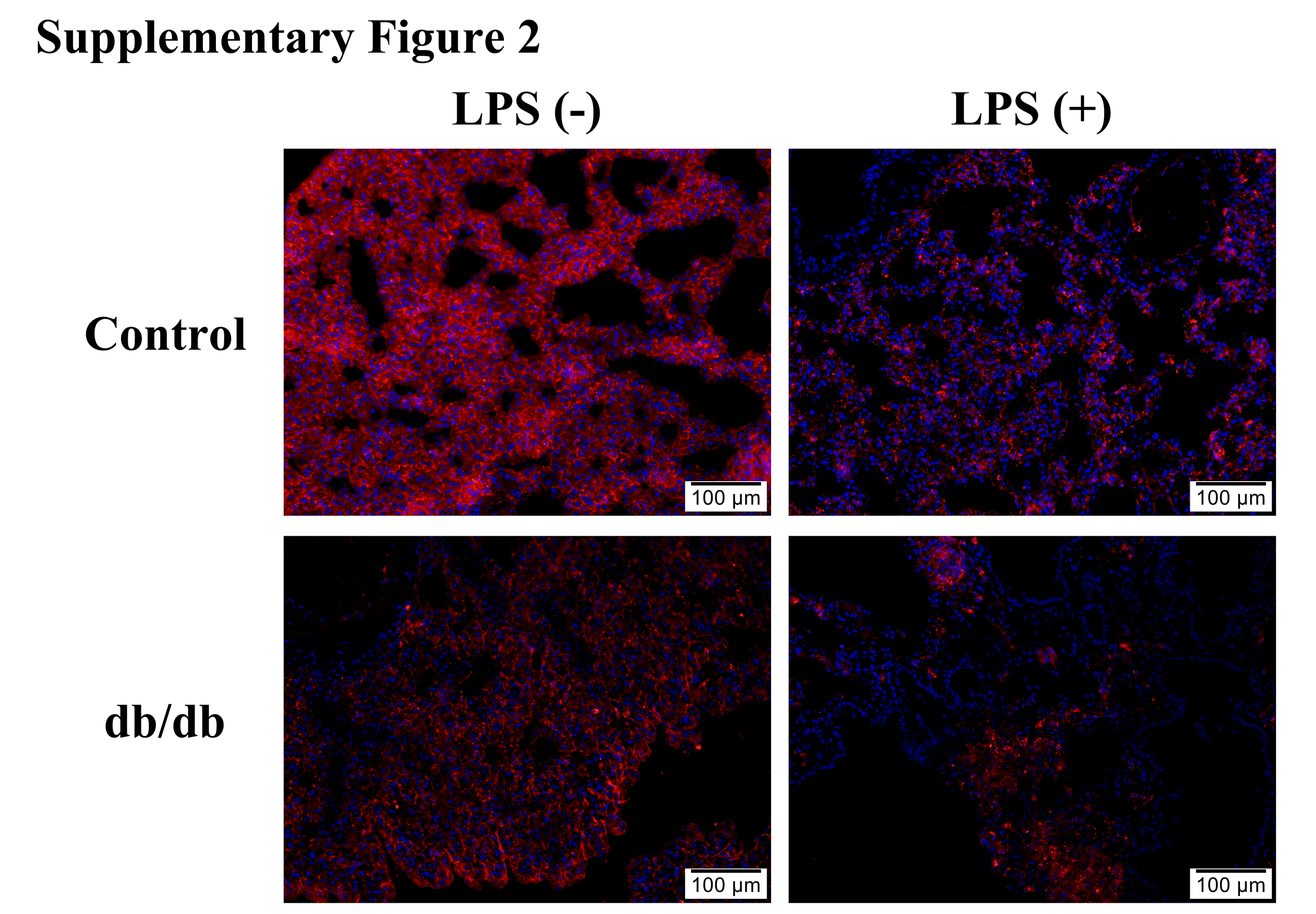

Supplement: Supplementary file 2 [file Image_2.TIF]
