# Supplementary material for: Endothelial Glycocalyx Disorders May Be Associated With Extended Inflammation During Endotoxemia in a Diabetic Mouse Model
Source: Front Cell Dev Biol. 2021 Apr 1;9:623582. doi: 10.3389/fcell.2021.623582 (PMC8047120; doi:10.3389/fcell.2021.623582)
Supplement: Supplementary file 3 [file Table_1.DOCX]

**Supplementary Table 1: Primers for relative quantification Real time RT-PCR Analyses**

| ***SDC1*** | **F 5’ CAAGGAAAAGGAGGTCACCA 3’**  **R 5’ CTGATTGGCAGTTCCATCCT 3’** |
| --- | --- |
| ***Has1*** | **F 5’ TGAGACAGGACATGCCAAAG 3’**  **R 5’ CGATCTGAAGCCAGAGGAAC 3’** |
| ***Has2*** | **F 5’ ATAAGCGGTCCTCTGGGAAT 3’**  **R 5’ CCTGTTGGTAAGGTGCCTGT 3’** |
| ***Csgalnact1*** | **F 5’ GCCAGTGAGGAAAGACAAGC 3’**  **R 5’ GCCTTTATCCCTTTCGGTTC 3’** |
| ***Ext1*** | **F 5’ ATTATCGGGAAATGCTGCAC 3’**  **R 5’ TCCGTTGCTGAGCATTACAG 3’** |
| ***VCAN*** | **F 5’ TTCAGGCAGCTTGGAGAAAT 3’**  **R 5’ TACAGGGTTCTCACCCCAAG 3’** |
| ***GAPDH*** | **F 5’ ATGTTCCAGTATGACTCCACTCACG 3’**  **R 5’ GAAGACACCAGTAGACTCCACGACA 3’** |
